# Degenerate T-cell Recognition of Peptides on MHC Molecules Creates Large Holes in the T-cell Repertoire

**DOI:** 10.1371/journal.pcbi.1002412

**Published:** 2012-03-01

**Authors:** Jorg J. A. Calis, Rob J. de Boer, Can Keşmir

**Affiliations:** Theoretical Biology & Bioinformatics, Utrecht University, Utrecht, The Netherlands; La Jolla Institute for Allergy and Immunology, United States of America

## Abstract

The cellular immune system screens peptides presented by host cells on MHC molecules to assess if the cells are infected. In this study we examined whether the presented peptides contain enough information for a proper self/nonself assessment by comparing the presented human (self) and bacterial or viral (nonself) peptides on a large number of MHC molecules. For all MHC molecules tested, only a small fraction of the presented nonself peptides from 174 species of bacteria and 1000 viral proteomes (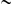
0.2%) is shown to be *identical* to a presented self peptide. Next, we use available data on T-cell receptor-peptide-MHC interactions to estimate how well T-cells distinguish between similar peptides. The recognition of a peptide-MHC by the T-cell receptor is flexible, and as a result, about one-third of the presented nonself peptides is expected to be *indistinguishable* (by T-cells) from presented self peptides. This suggests that T-cells are expected to remain tolerant for a large fraction of the presented nonself peptides, which provides an explanation for the “holes in the T-cell repertoire” that are found for a large fraction of foreign epitopes. Additionally, this overlap with self increases the need for efficient self tolerance, as many self-similar nonself peptides could initiate an autoimmune response. Degenerate recognition of peptide-MHC-I complexes by T-cells thus creates large and potentially dangerous overlaps between self and nonself.

## Author Summary

Human cells sample short peptides from endogenous proteins, and present them to the immune system via HLA class I molecules on the cell surface. T-cells scan the presented peptides and need to discriminate foreign (nonself) peptides from human (self) peptides. We show that this is a difficult task, despite the exquisite specificity of T-cells. We estimate, using HLA-peptide binding predictions and T-cell recognition models, that almost a third of the nonself peptide-HLA complexes is so similar to a self peptide-HLA that a T-cell cannot tell them apart. Since T-cells have to ignore self peptides to prevent autoimmunity, we estimate that at least a third of the foreign peptides has to be ignored as well, and therefore fails to evoke an immune response. Foreign peptides that are never used in immune responses, have been referred to as the “holes in the repertoire”. Since the sizes of the holes we predict agree with those that were previously found, our conjecture is that the holes are entirely due to similarity with self peptides. We test this conjecture with public data on HIV-1 and vaccinia responses, and confirm that self similarity is a major determinant of the immune response to nonself peptides.

## Introduction

The recognition of peptide-MHC-I complexes (pMHC) by the T-cell receptor (TCR) is required for effector T-cells to kill an infected cell. Although some MHC-I molecules have a preference to present pathogen-derived peptides [1], pMHC are formed with both self and nonself peptides. Therefore, to allow CD8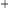 T-cells of the cellular immune system to discriminate self from nonself, presented nonself peptides should be different from presented self peptides. What would happen if a nonself peptide is so similar to a self peptide that it is recognized by the same T-cell (we will call such peptides “overlapping peptides”)? Firstly, an effector T-cell response to an overlapping peptide, could cause T-cell mediated autoimmune disease, such as type 1 diabetes [2]–[4] or multiple sclerosis [5], [6]. Secondly, to avoid autoimmunity, T-cells recognizing self-pMHCs are tolerized during negative selection [7]. Due to this self tolerance, overlapping nonself peptides should fail to elicit a T-cell response, and this may limit the number of pathogen-derived peptides that are available for an immune response and hence the chance to control a pathogen [8], [9]. Assarsson et al. showed that 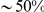 of the MHC-I presented vaccinia derived peptides are not recognized by T-cells [10]. Similarly, for HIV-1-derived peptides predicted to be presented on the well-studied HLA-A*0201 molecule, only 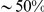 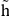
as been reported to elicit a T-cell response [9]. Taken together, these studies suggest large “holes” in the T-cell repertoire [8], [11], which could be caused by overlaps with self pMHCs.

We have previously shown that on HLA-A2 molecules only a minute fraction (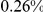) of the presented nonself peptides are identical to presented self peptides [12]. Such a small overlap cannot cause the large holes in the T-cell repertoire. However, at that time there was too little data available on T-cell recognition of pMHCs, to study its impact on the self/nonself overlap. It is well established that T-cells are cross-reactive and can recognize similar, and sometimes even unrelated, peptides presented on the same MHC molecule [13]. The principles of TCR-pMHC interactions that allow for this flexibility are not fully understood. CTL recognition-studies using peptide libraries with altered peptide ligands [9], [14]–[18] and pMHC-TCR structures [19], [20] allow some inferences to be made. The middle (P4–P6) part of the peptide forms the core of the interaction [9], [14]–[20], where the majority of amino acid substitutions (with exception of those with very similar amino acids) tend to perturb pMHC recognition. Other positions in the peptide, although not in direct contact with the TCR, can still be important for the TCR-pMHC interaction if they affect the configuration of the P4–P6 residues [14], or MHC-binding [21]. In most cases, the N-terminal position (P1) of the peptide is unimportant for the TCR-pMHC interaction [9], [14], [16]–[20].

Given these new insights, we here extend our previous investigations on self/nonself overlaps by including the T-cell recognition of pMHCs. In addition, we analyze the self/nonself overlap of peptides presented on several HLA-A and HLA-B molecules, to estimate the degree of variance among different MHC-I molecules. Using high-quality predictors of the MHC-I presentation pathway [22]–[25], we show that presented peptides derived from nonself are in almost all cases (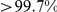) distinct from presented self peptides, for all common MHC molecules. This result is in agreement with our original observation that most peptides with a length of nine amino acids (9 mers) of unrelated species are unique [12]. However, the cross-reactivity of T-cell recognition is shown to increase the self/nonself overlap between sufficiently similar peptides to about one-third. Our results suggest an explanation for the observed holes in the T-cell repertoire during an infection, and we show that our self/nonself overlap estimates can be used to distinguish immunogenic from non-immunogenic pMHCs. Moreover, the estimates of self/nonself overlap demonstrate that the risk of autoimmunity due to molecular mimicry with pathogens is nonnegligible.

## Results

### Self/nonself overlaps based on peptides

**Figure 1:**
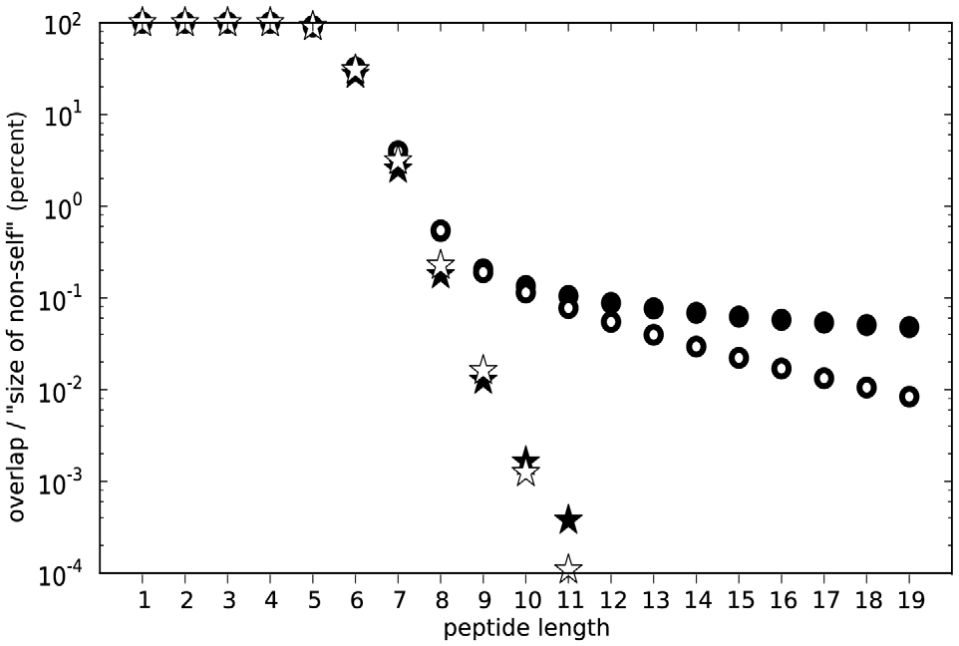
Viral and bacterial self/nonself overlaps for peptides of different lengths. The chance that a bacterial or viral peptide overlaps with a peptide in the human proteome is shown as open and closed circles for bacteria and viruses, respectively. Stars indicate the self/nonself overlaps with shuffled bacterial (open stars) or viral (closed stars) proteins. For all peptides of 5 amino acids or longer, the overlap of unshuffled viruses and bacteria is significantly smaller than the shuffled (representing the expected) overlap (Ranksums test: p
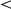
0.05).

MHC class I molecules shape CD8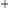 T-cell responses via the presentation of peptides derived from intracellular proteins. These peptides are short: most MHC-I molecules prefer to bind peptides of 9 amino acids (9 mers). To investigate how similar self and nonself peptides are, the human and a large number of nonself proteomes (data selection is detailed in Methods) were cut into fragments of various lengths (1–20 amino acids long) and peptides that occur both in self and nonself proteomes were identified (i.e. without considering MHC-I presentation). The fraction of foreign peptides that are also present in the human proteome defines the “overlap”, i.e. the chance that a randomly chosen nonself peptide is identical to a self peptide. For small peptides shorter than five amino acids, the overlap is 100%, since almost every 5mer is present in the human proteome (see Figure 1). For longer peptides the overlap decreases rapidly, and at a length of 9 amino acids the average overlap is only 0.20% for viruses (between 0–0.5% for 95% of all viruses) and 0.19% for bacteria (0.1–0.4% for 95% of all bacteria). These results are in excellent agreement with our previous estimates based on a much smaller set of nonself proteomes [12]. To conclude, 9 mers contain enough information to discriminate self from nonself, i.e. the chance that a nonself 9mer overlaps with a self 9mer is only 0.2%.

Surprisingly, the overlaps do not decrease much further for peptides longer than 9 mers (see Figure 1). To characterize these overlapping sequences further, for each human protein we counted the number of viruses or bacteria that has at least one overlapping 9mer peptide. The proteins where this number was larger than expected (p
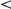
0.01, see Methods) were analyzed by a functional annotation cluster analysis [26], [27]. This analysis showed that bacterial 9 mers tend to overlap with human proteins of mitochondrial origin, which is in line with the bacterial origin of mitochondria [28]. In addition, proteins involved in metabolic processes that might be common to bacteria and humans had more overlapping 9 mers (see Table S1). For viruses, the overlap is largest with nuclear proteins and transcription factors that are possibly acquired via horizontal gene transfer to modulate host cellular processes (see Table S1). In order to test the effects of homologous sequences or convergent evolution on self/nonself overlaps, sequences were shuffled before examining the overlap to break up any overlap that might be the result of these effects. Indeed, this shows that a far majority of the overlaps were due to these homologous sequences as the overlaps in shuffled sequences are much lower than the actual overlaps (Figure 1, in stars).

### Self/nonself overlaps based on peptide-MHC-I complexes

Only peptides that are presented on an MHC-I molecule, i.e. about 1–3% of all 9 mers [10], can be recognized by T-cells. Due to the binding preferences of different MHC-I molecules, the self/nonself overlap of MHC-I presented peptides can be different per MHC-I molecule and does not need to be the same as the overlap based on all 9 mers. For instance, we recently showed that certain MHC-I molecules have a preference for pathogen-specific peptides [1]; such a preference should decrease the self/nonself overlap for that MHC-I molecule. To estimate the self/nonself overlap of MHC-I presented peptides, an *in silico* approach was undertaken using state-of-the-art MHC-I pathway predictors [22]–[25] (see Methods).

**Figure 2:**
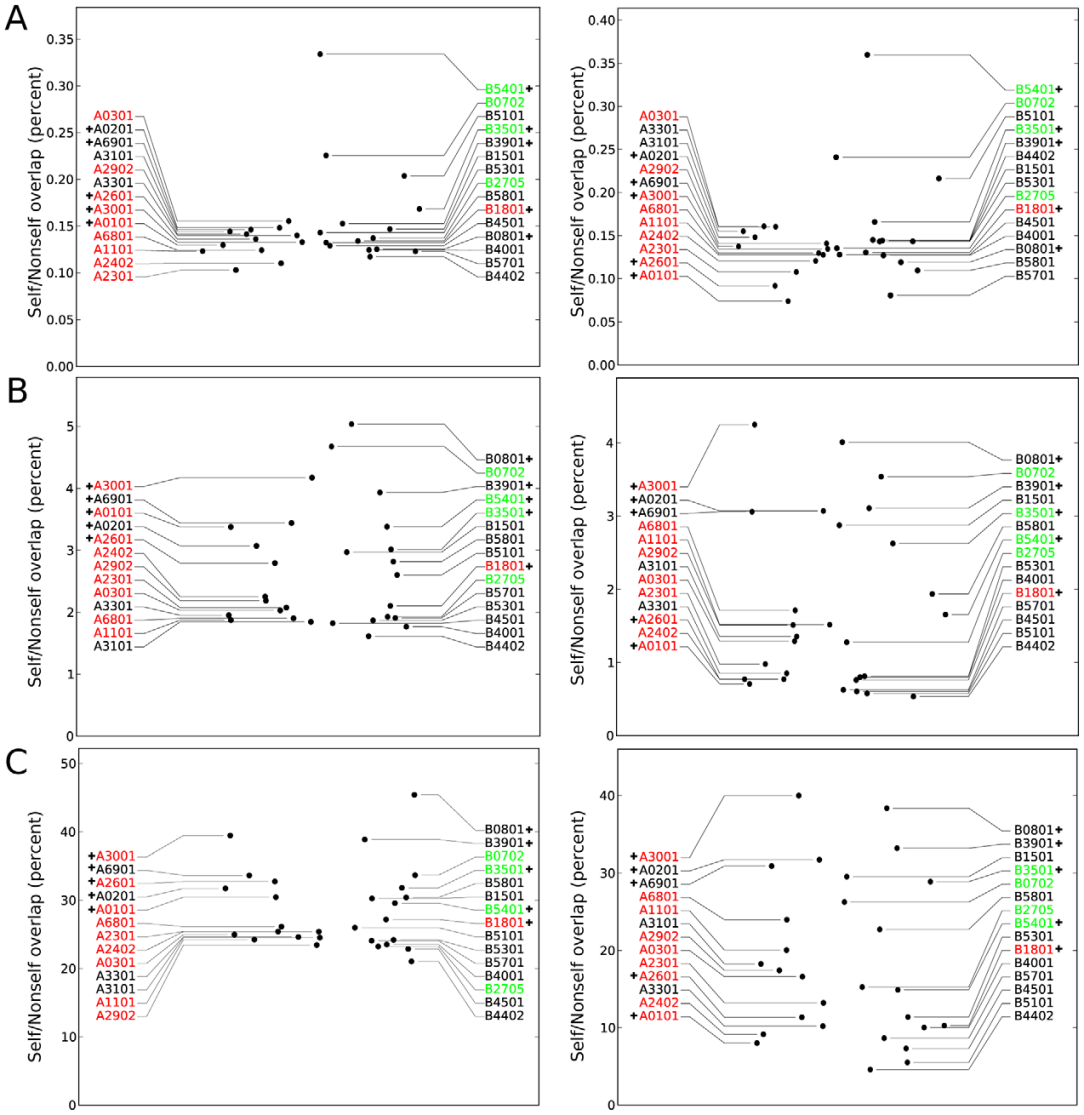
Self/nonself overlaps of peptides presented on different HLA molecules. In A, the exact overlap of the complete peptide (positions 1–9). In B, the exact overlap of the middle positions of the peptide (positions 3–8) that are assumed to be in contact with the TCR. In C, the degenerate overlap of positions 3–8, i.e. a cross-reactive T-cell overlap. In all cases, the left and right figures show the self/nonself overlaps determined using a scaled or fixed MHC binding threshold, respectively (see Methods). HLA molecules that have been described to have a GC-positive, GC-negative or GC-neutral preference [1] are colored green, red and black, respectively. HLA molecules with additional anchors (see Methods) are indicated with a plus-sign.

For a large set of common human MHC-I molecules (13 HLA-A molecules and 15 HLA-B molecules, see Methods for selection criteria), the presented peptides in the human proteome and a large set of nonself proteomes were predicted. To define presented peptides we made use of the well-studied HLA-A*0201 molecule. For this molecule an IC50 value of 500 nM is often taken as threshold to separate the binders from non-binders. Applying this threshold to all self peptides we find that HLA-A*0201 has a specificity of 2.3%, i.e. 2.3% of the tested peptides would be binders. For other HLA molecules we determined “scaled” binding thresholds, so that they have the same specificity as HLA-A*0201, i.e. they present 2.3% of all self peptides. Next, the overlap between presented self and nonself peptides was enumerated per MHC-I molecule, by comparing for each HLA molecule, self and nonself peptides presented on that HLA molecule. On average, only 0.15% of the MHC-I presented nonself peptides is identical to a presented self peptide (see Figure 2A, left). The average overlap of MHC-I presented peptides is somewhat smaller than the overlap of all 9 mers in the proteome (0.2%, see Figure 1), which is in agreement with the fact that many MHC-I molecules have a slight preference for pathogen-derived peptides [1]. The maximal overlap of 0.33%, which is still very low, was found for peptides presented by HLA-B*5401. These results demonstrate that for all common human MHC-I molecules, only a minute fraction of the presented nonself peptides is identical to a presented self peptide. By using scaled binding thresholds, we take the conservative assumption that different HLA molecules have similar specificities, this does not have to be so. The self/nonself overlaps were also calculated by using a fixed binding threshold of 500 nM, which leads to different specificities for different HLA molecules. In this case, the self/nonself overlap determined for peptides presented on different HLA molecules remained as low as when scaled thresholds were used (see Figure 2A, right).

### Self/nonself overlaps based on T-cell recognition

So far, we only considered identical self and nonself peptides as overlaps. However, also non-identical MHC-I presented peptides can be recognized by the same T-cell [13]. This cross-reactivity is partly due to the fact that not all the residues on a presented peptide are accessible for the TCR. For example, most MHC-I molecules have two binding pockets that bind positions 2 and 9 (i.e. anchor-residues) of the presented peptide. These anchor-residues are hidden in the binding pocket of an MHC-I molecule, and are not exposed to the TCR [29]. Recently, we analyzed the T-cell recognition of the HIV-1 derived SLFNTVATL peptide presented on HLA-A*02 and suggested that not only the anchor-residues (P2 and P9), but also the first position (P1) of the presented peptide, hardly affects T-cell recognition [9]. Furthermore, at the remaining six middle positions (P3–8), some amino acid substitutions did not perturb T-cell recognition, especially those between amino acids with similar physical-chemical properties. TCR recognition was most stringent at the fifth position (P5), where only a Threonine-to-Serine substitution did not affect recognition [9].

**Figure 3:**
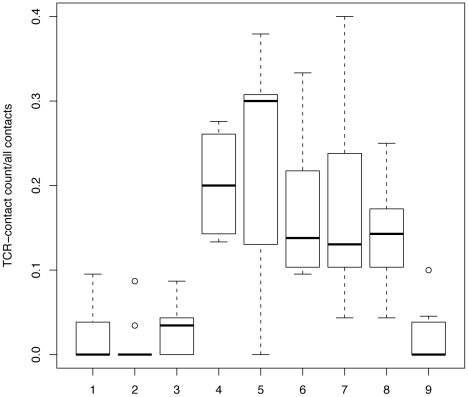
TCR interactions per peptide position. TCR contacts for 9 pMHC-TCR structures that have a 9mer (see Methods for details on selection and analysis criteria) were determined per position of the peptide. Per position the fraction of TCR-contacts relative to the total number of peptide-TCR contacts in a structure is shown. Positions 4–8 all have a significantly higher number of interactions than positions 1–3 and 9 have (Ranksums test: p
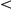
0.005).

To see if other TCR-pMHC contacts follow the same interaction-“rules”, all non-redundant TCR-pMHC-I structures found in the PDB-database (www.pdb.org [30]) encompassing a 9mer (n = 9, see Methods for selection criteria) were studied. In agreement with Frankild et al. [9], the majority of interactions in these structures involved the middle positions of the presented peptide (Figure 3). Several other reports on TCR-pMHC structures, and on different T-cell clones, confirm the degeneracy at the first position, and confirm that substitutions among similar amino acids are allowed in other positions [14]–[20]. Our structural analysis suggests that the third position has less contacts with the TCR than the other middle positions (Figure 3). However, Tynan et al. [14] show examples in which position 3 is important for T-cell recognition. Therefore, we conservatively assume that the third position is as important for T-cell recognition as the other middle positions (P4–8).

Given these data, we studied how much of presented nonself can be discriminated from presented self by T-cells. First, the self/nonself overlaps were determined on those positions recognized by T-cells, i.e. the middle positions (P3–8) of MHC-I presented peptides. The self/nonself overlap of these 6mer fragments is on average 18 times higher than the overlap based on all positions (i.e., 2.7% for scaled thresholds and 1.7% for fixed thresholds see Figure 2B). This increase in the overlaps is mainly due to excluding the first position: if only both anchor positions are discarded, the overlap determined on the non-anchor positions (P1 and P3–8) remains low (i.e. 0.4% on average, see Table 1 and Figure S1). Similarly, if only one of the anchor positions and position P1 are discarded, the overlap is much higher (Table S2). We showed previously that highly specific anchor-positions of MHC molecules do not have to be exposed to the TCR to contribute to self/nonself discrimination because T-cells are MHC restricted [12]. For instance, HLA-A*0101 has a very specific preference for Tyrosine at the second anchor position (P9), and even if an HLA-A*0101 restricted T-cell is not interacting with this amino acid, all presented peptides it can possibly respond to must have a Tyrosine at position 9.

**Table 1:** Summary of all the average self/nonself overlaps obtained using peptides predicted to be presented on HLA molecules.

		Recognized peptide positions		
	Self	P1–9	P1 and P3–8	P3–8
	percentage	(complete)	(non-anchor)	(middle)
Exact	100	0.15%*	0.41%	2.7%*
	50	0.09%	0.25%	1.6%
Degenerate	100	0.7%	5.2%	29%*

Overlaps were determined using all positions of the peptide (P1–9), the non-anchor positions (P1 and P3–8) or the middle positions between the anchors (P3–8). Further, overlaps were determined as exact, i.e. every position should be identical, or as degenerate, i.e. with 1 or 2 substitutions being allowed to mimic T-cell recognition (see Methods). Finally, overlaps with 100% or (a randomly chosen) 50% of the human proteome are shown. Self/nonself overlaps indicated with a star (*) are shown per HLA molecule in Figure 2.

Next, overall self/nonself overlaps were estimated with a novel model of degenerate T-cell binding. As above, T-cells were assumed to bind to the middle positions (P3–8) of the MHC-I presented peptides only. In addition, the degeneracy was modeled by considering two peptides as overlapping if they have mismatches in maximally two regions. We allow one mismatch at the N-terminal side of the fifth position (P1–4) and one at the C-terminal side of that position (P6–9) (see Methods). Moreover, only mismatches between amino acids having similar peptide-protein interaction properties were allowed, as such conservative substitutions have been shown to have a limited influence on T-cell recognition [9], [13]–[15]. The similarity between amino acids was derived from the PMBEC amino acid substitution matrix, that is based on peptide-MHC interactions and therefore specifically tailored to estimate the influence of amino acid substitutions on peptide-protein interactions [31]. We refer to this new overlap as the “degenerate” overlap. The degenerate self/nonself overlap is much higher than the identical overlaps of P3–8, on average 29% (see Figure 2C, left). These results can be ascribed to the degenerate nature of T-cell recognition: when using an alternative model of TCR recognition described by Frankild et al., the “peptide similarity score”-method (see Methods) [9], similarly high self/nonself overlaps were observed (results not shown). The self/nonself overlaps based on middle positions of the presented peptide (P3–8), determined using fixed binding thresholds were very similar to the overlap based on scaled thresholds (see Figures 2C, right), though more varied and somewhat lower. This is a result of the differences in the specificities of HLA molecules. The specificity determines the fraction of presented self and nonself peptides, which in turn influences the chance of finding a self/nonself overlap. One can explain this intuitively as the following: if an MHC molecule is very specific, it presents a small set of self peptides. For every presented nonself peptide, the chance of having an overlap with self would then become smaller. Therefore, there is a strong correlation between binding specificity and self/nonself overlaps (see Figure S2). Furthermore, we tested the robustness of our results for various methods of peptide binding predictions, measures of amino acid similarity, and assumptions on T-cell recognition (summarized in Table S2). In all cases did degenerate T-cell recognition lead to a high self/nonself overlap of 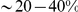.

Despite the high overlaps, our assumptions on the degenerate T-cell recognition can be considered conservative. For example, position 3 of the presented peptide tends to have few interactions with the TCR (see Figure 3) and our model should probably allow more mismatches at this position. Furthermore, many peptides with more than two substitutions at the middle positions (P3–8) have been shown to be cross-reactive [9]. If we assume that only a fraction of the self proteins provides a source of presented peptides, our estimates on self/nonself overlap decrease proportionally (see Table 1 and Table S2). Cole et al. [21] recently showed that in some cases, the anchor residues are involved in T-cell recognition. This observation might be more of an exception rather than the general mode of T-cell recognition, as in most cases T-cell recognition has been described to be less specific and not influenced by the anchor residues [9], [13], [14], [19], [29]. Recent estimates on T-cell crossreactivity confirm that our model remains conservative. Ishizuka et al. tested the T-cell recognition of 30.000 unrelated MHC-I presented peptides using human and Murine T-cell clones, and found a single cross-reactive response, which suggested a cross-reactivity level of 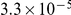 (1/30000) [32]. Typical T-cell precursor frequencies in a mouse are 1/100000 [33]–[35], i.e. on average 1 in a 100.000 T-cells are expected to recognize a particular pMHC, and 1 in a 100.000 pMHCs are expected to be recognized by a single T-cell clone. In other words, precursor frequency and cross-reactivity are similar concepts reflecting the specificity of a T-cell [36]. In our degenerate T-cell recognition model, single T-cells recognize only one in 2.7 million (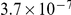) pMHCs (see Methods). Since this is much more specific than the experimental estimates, we think that our degenerate self/nonself overlap of about one-third is conservative and underestimates the actual overlap.

### Consequences of a high self/nonself overlap

Although these estimates on cross-reactive overlaps remain relatively crude, our results show that the degenerate recognition of MHC-I presented peptides by T-cells has a profound effect on self/nonself discrimination. This reconfirms that deletion of self reactive T-cells is important, as many of them would be activated during an infection and induce an autoimmune response. As a consequence, we estimate that about a third (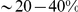) of the foreign pMHCs is expected not to trigger an immune response. To test this prediction, the self/nonself overlap of HIV-1 derived peptides presented on HLA-A*0201 was studied to see if our model can account for the observed poor immunogenicity of these peptides. The presentation of, and T-cell responses to, HIV-1 derived peptides presented on HLA-A*0201 has been the subject of extensive investigations. Because it is such an intensively studied system, the lack of a reported T-cell response for one of the predicted pMHCs can be used as a reasonable indication for the lack of immunogenicity of that pMHC [9]. One explanation for the lack of immunogenicity is an overlap of the epitope with a self pMHC, and hence the self tolerance of the corresponding T-cell clone. We tested this by comparing overlaps of immunogenic and non-immunogenic HIV-1 pMHCs with self (see Methods). Only 4 of the 33 immunogenic pMHC (12%) were found to overlap with self according to our degenerate T-cell recognition model using the PMBEC similarity matrix. A significantly higher fraction of non-immunogenic pMHC, i.e. 18 of 54 (33%), overlapped with self (Chi-square test: p = 0.027) (see Table 2), which is comparable to the overlaps reported by Frankild, using a different model for self-similarity but the same pMHCs [9]. We extended the analysis of self/nonself overlaps to vaccinia-derived peptides presented in HLA-A*02-transgenic mice for which Assarsson et al. [10] have determined the immunogenicity (see Methods). The overlap between (murine) self and immunogenic peptides is again lower than the self overlap of non-immunogenic peptides, although not significant due to the small number of data points (see Table 2). These results are also valid for other HLA molecules: using data provided by Perez et al. [37] on non-HLA-A*0201 presented HIV-1 peptides we found the same trend, that immunogenic peptides have less self/nonself overlaps than their non-immunogenic counterparts (see Table 2, and Methods). Finally, we analyzed immunogenic/non-immunogenic pMHCs derived from the IEDB [38] that were presented on the same HLA molecule (see Methods for selection criteria). The number of immunogenic and non-immunogenic pMHCs was large enough only for HLA-A*0201, and therefore the self/nonself overlaps of these sets were compared. Again, we found significantly less self overlaps among immunogenic peptides than non-immunogenic ones (Chi-square test: 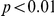; see Table 2). These results on the HLA-A*0201 presented HIV-1 and IEDB peptides are robust to the model assumptions: In all alternative overlap models described in Table S2, the number of overlaps with self was smaller for immunogenic pMHCs than for non-immunogenic pMHCs. This difference was always significant for the large set of IEDB peptides, for the smaller set of HIV-1 peptides a significant difference was not always observed (data not shown). Thus, in various data sets and model assumptions we find a correlation between pMHCs being immunogenic and their overlap with self, but these correlations only become significant for HLA-A*0201 where there is enough data. Summarizing, high self/nonself overlaps can explain the observed large “holes” in the T-cell repertoire [8], [11], and play an important role in determining the immunogenicity of foreign pMHCs.

**Table 2:** The self/nonself overlap of immunogenic versus non-immunogenic pMHCs.

	Immunogenic		Non-Immunogenic		Chi 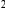 -test (p-value)
	Self Overlapping	Not Overlapping	Self Overlapping	Not Overlapping	
HIV-1 peptides on HLA-A*0201	4	29	18	36	0.027
Vaccinia peptides on HLA-A*0201	3	15	8	18	0.29
HIV-1 peptides on non-HLA-A*0201 molecules	0	9	4	9	0.066
HLA-A*0201 pMHC from the IEDB	54	143	230	362	0.0038

For immunogenic or non-immunogenic HIV-1 peptides presented on HLA-A*0201 determined by Frankild et al. [9], for immunogenic and non-immunogenic vaccinia-derived peptides determined by Assarsson et al. [10], for immunogenic and non-immunogenic HIV-1 peptides on non-HLA-A*0201 determined by Perez et al. [37] and for immunogenic and non-immunogenic pMHCs sampled from the IEDB on HLA-A*0201 (see Methods for selection criteria applied to all four data sets), the presence of a self/nonself overlap was determined with the degenerate T-cell recognition model. For all sets of peptides, the immunogenic peptides have less overlaps with self, the significance of this association was tested using a Chi-square test, the p-value is reported in the last column.

## Discussion

Previously, we have shown that the few epitopes sampled from a pathogens proteome are likely to be unique and are not expected to be present in the host (human) proteome [12]. Here, we extend this study by investigating a much larger set of nonself proteomes and a larger set of common HLA molecules. From this analysis we conclude that the pMHC of all common HLA-A and HLA-B molecules carry enough information for self/nonself discrimination, as a small minority (0.1% to 0.3%) of nonself derived peptides is expected to be identical to presented self-peptides. However, if the degenerate T-cell recognition of pMHCs is taken into account, the results change drastically. The cross-reactive recognition by T-cells results in a much higher self/nonself overlap of 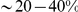 that is robust to various assumptions on degenerate T-cell recognition (see Table S2), i.e. in the “eyes” of a T-cell, about a third of the epitopes is expected to be similar to a self peptide presented on the same MHC-I molecule. Such a large overlap is expected to have a strong effect on the immunogenicity of pathogen-derived epitopes.

One might intuitively think that the high self/nonself overlap estimates are in disagreement with the exquisite specificity of T-cell recognition. However, in our “degenerate” model of the middle positions (P3–8) with maximally 2 conservative mismatches, an individual T-cell recognizes only one in 2.7 million pMHCs. This level of specificity is much higher than experimental measurements of about one in 100.000 [32]–[35]. Therefore, we think that our current self/nonself overlap estimates are conservative.

Could longer peptides be a solution for the high self/nonself overlaps caused by degenerate T-cell recognition? Given that T-cells cannot use all the information that is present in an MHC-I presented 9mer, we do not expect that the presentation of longer peptides would make much difference. Even though a longer peptide would contain more information, if that is not detected by the T-cells it would not improve self/nonself discrimination. Alternatively, MHC binding could be more specific at for instance position 1, thus preserving self/nonself information as now happens at the anchor positions. The disadvantage of more specific binding motifs would be the reduced presentation of foreign peptides and more opportunities for a virus to escape MHC presentation.

Another consequence of a high self/nonself overlap could be high risk of autoimmunity. The identification of self antigens targeted in autoimmune diseases remains an enormous challenge, and our method of identifying overlapping peptides could possible help to narrow the search for these auto antigens. This requires a thorough understanding of the pathogens that might trigger a particular autoimmune disease and the corresponding HLA risk factors. Unfortunately, only for few autoimmune diseases sufficient data is available to extract such associations. For instance, Epstein Barr virus and HLA-B*4402 are associated with multiple sclerosis [39], [40], and HTLV-1 and HLA-B*5401 are associated with HAM/TSP [41]. We are currently searching the overlaps between the presented peptides of these viruses and the human self peptides presented on these HLA molecules for potential CTL targets in these autoimmune diseases (work in progress).

The predicted self/nonself overlap varies between HLA molecules (see Figure 2), and two factors explain most of this variation. First, some HLA molecules have a preference for peptides derived from organisms with a low G+C content [1], which seems to be a universal signature for pathogenicity [42]. HLA molecules with such a preference for presenting nonself (e.g. HLA-A*2301) have a lower self/nonself overlap than other HLA molecules, because they present peptides that are less likely to occur in the human proteome. Second, the usage of additional (auxiliary or atypical) anchors at positions that also interact with the TCR increases the chance that presented peptides overlap according to our model. For example, HLA-B*0801 with atypical anchors at the third and fifth position will present more peptides that overlap at position three and five, and has the highest estimated self/nonself overlap (see Figure 2C). Indeed, a strong correlation between the use of additional anchors (see Methods) and self/nonself overlaps is found (Spearman Rank test: correlation = 0.88, 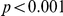, not shown). Possibly, peptides presented on HLA-B*0801 have more specific TCR-interactions at the conventional anchor positions (P2 and P9) than in our T-cell recognition model, leading to an overestimate of the self/nonself overlap for this HLA molecule and others with atypical anchors. If the degenerate self/nonself overlap is not based on the middle positions of the presented peptide (P3–8), but on an HLA molecule specific choice of the six least specific positions (see Methods), the overlaps are however very comparable to an overlap based on the middle positions (see Table S2).

Our estimates on self/nonself overlaps can explain why MHC-I restricted cellular immune responses to a pathogen are more narrow than the (predicted) number of pMHCs for that organism [9], [10]. We show that about one-third of the nonself pMHC should not elicit T-cell responses because they overlap with a self pMHC, i.e. this explains the large “holes” found in the T-cell repertoire [8], [9], [11]. We validated this prediction by comparing the overlaps of immunogenic and non-immunogenic pMHC from HIV-1, vaccinia or the IEDB, and showed that the number of self overlaps is significantly higher for non-immunogenic pMHC than for immunogenic pMHC. Still, a fraction of the immunogenic pMHCs were predicted to be overlapping with self, possibly because not all self-proteins induce tolerance or because regulatory processes are overridden during some viral infections causing autoimmunity [43]. In addition, an improved understanding of the rules of T-cell recognition could result in an even better distinction between overlapping/non-overlapping, and non-immunogenic/immunogenic pMHCs. This would be important in vaccine design and the understanding of immunogenicity in cellular immune responses.

## Methods

### Proteome data collection

Human, Murine, viral and bacterial proteomes were downloaded via http://www.ebi.ac.uk, the human proteome in May 2008, bacterial and viral proteomes in October 2008 and the Mouse proteome in January 2011. Only human and mouse proteins that have been shown at the protein or transcript level were included in the “self” data set. Redundant bacterial proteomes were removed by selecting only one strain per species, which resulted in 174 species of bacteria. 1000 non-redundant viral proteomes were selected with a maximum similarity of 80%. The similarity between viruses was determined as the number of exact matches in an all-to-all alignment of proteome sequences using BLASTP 2.2.18 relative to the smallest virus. Human viruses were selected based on the reported host information in the downloaded proteome, or on the term ‘human’ in their species name (e.g. Human Immunodeficiency Virus). A list of all bacteria and viruses used in this study is available upon request.

### MHC-I presentation predictions

The peptides presented on a certain MHC-I molecule can be predicted by simulating three key-processes of MHC-I presentation, i.e. proteasomal cleavage, TAP transport and peptide-MHC-I binding. The combination of proteasomal cleavage and TAP-transport determines which peptides reach the ER to potentially bind MHC-I. This process was predicted using NetChop Cterm3.0 [22], [23]. Peptide-MHC-I binding was predicted using NetMHC-3.2, an improved version of NetMHC-3.0, that was shown to perform best in a large benchmark study of Peters et al. [24], [25]. The fraction of nonself peptides that overlap with a self peptide presented on an MHC-I molecule depends on the number of self peptides that is predicted to bind to this MHC-I molecule. Because we want to compare the self/nonself overlap of different MHC-I molecules, we have chosen to exclude the variance in the number of presented self peptides by using scaled thresholds, i.e., the number of self peptides predicted to bind to each MHC molecules is scaled to be similar. Unfortunately, this procedure will eliminate the variation as a result of possible differences in specificity among MHC molecules. For each MHC molecule the threshold was set such that the presented fraction of self was similar to that on HLA-A*0201 with a 500 nM threshold (2.3%) [44], [45]. This results in on average 250.492 self pMHCs, 3.750.428 bacterial and 196.265 viral pMHCs, per HLA molecule. Alternatively, we repeated the analysis with a fixed threshold of 500 nM (see Figure 2 and Table S2). In order to exclude HLA molecules with too similar binding motifs from our analysis, we selected the most frequent HLA molecule available in NetMHC-3.2 at two digit resolution. This resulted in a set of 13 HLA-A and 15 HLA-B molecules.

All results were checked for consistency with two other MHC-I binding prediction methods, NetMHCpan-2 [46] and a Stabilized Matrix Method (SMM)-based MHC-binding prediction tool [47], for HLA-A*0101, HLA-A*0201, HLA-A*0301, HLA-B*0702, HLA-B*0801 and HLA-B*3501. Note that for the HLA molecules that we have included in our analysis the average AUC for NetMHC and NetMHCpan predictions is 0.809 and 0.812, respectively [48]. As expected, similar results were obtained with NetMHCpan, but also when using SMMs (Table S2).

### Self/nonself overlap estimations

Per MHC-I molecule, the set of presented 9 mers derived from viral or bacterial (nonself) proteomes and that from the human (self) proteome were compared to see how much these sets overlap. In the self/nonself overlap determination for vaccinia-derived pMHC from Assarsson et al. [10], the Mouse proteome was used as self. Overlaps were determined in different ways. First a “complete overlap” was determined as the exact match of all positions of the 9mer (positions 1–9, as in Figure 2A). Second, a “middle positions 6mer overlap” was defined as an exact match of the amino acids at positions 3–8 (as in Figure 2B). Third, the “non-anchor 7mer overlap” was determined as the exact match of the amino acids at position 1 and 3–8 (as in Figure S1). Finally, a “degenerate overlap” was determined by allowing two amino acid mismatches. Amino acid mismatches were not allowed at the most specifically recognized position 5. Moreover, we reasoned that two amino acid substitutions close-by would be more likely to abolish T-cell recognition. Therefore, only a single mismatch was allowed at the positions N-terminal from position 5 (P1–P4) and at the positions C-terminal (P6–P9) from position 5. Finally, only mismatches between amino acids with similar peptide-protein interaction properties were allowed. Following Kim et al., amino acids were considered similar if their absolute covariance was greater than 0.05 in the PMBEC matrix [31]. The PMBEC matrix is based on measured binding affinities between peptides libraries and MHC-I molecules, and was shown to capture similarity features common to substitution matrices such as BLOSUM50, and outperform other matrices when used as a Bayesian prior in MHC-I binding predictor training [31]. Furthermore, repeating our analysis using a positive score in the BLOSUM62 or BLOSUM50 matrix to identify allowed mismatches, similar results were found (Table S2). The self/nonself overlap is the chance a nonself pMHC overlaps with self, and was calculated by dividing the total number of overlaps in all nonself proteomes by the total number of pMHCs in all nonself proteomes. The self/nonself overlap was determined for bacteria and viruses separately, and the average of these two self/nonself overlaps is presented throughout the paper.

Additionally, self/nonself overlaps were estimated using the “peptide similarity score”-method described in detail by Frankild et al. [9]. In this method the similarity between two peptides is determined using the BLOSUM35 amino acid substitution matrix and all positions of the compared peptides. The similarity score is subsequently scaled to the minimal and maximal similarity scores for the reference peptide, in order to normalize for the intrinsic similarity that a certain peptide has to all other peptides. If for instance the BLOSUM35 similarity score between peptide A and peptide B is 3, and the minimum and maximum possible similarities for any peptide with peptide A are 1 and 11, respectively, the peptide similarity score is 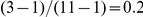 (see [9] for a full description of the method). Frankild et al. showed that a self similarity score of 0.85 tends to separate too self-similar, and hence non-immunogenic, from immunogenic HIV-epitopes [9]. This analysis and an analysis of cross-reactive peptides from literature was used for verification of this method [9]. We used the same threshold when determining overlaps with this “peptide similarity score”-method, i.e. nonself peptides with a similarity score exceeding 0.85 with a self peptide are considered as overlapping.

### Cross-reactivity

The cross-reactivity in our degenerate overlap model of T-cell recognition (described above) was determined in order to compare it with experimentally determined levels. For every possible 9mer peptide, the number of variants at the T-cell recognized middle positions (P3–8) was determined that would be recognized by the same T-cell in our degenerate overlap model. In other words, for every combination of amino acids at P3–8 we performed an exhaustive search to determine how many other combinations would also be recognized. On average, 24 of such combinations were found. Thus, given the number of possible variants at positions P3–8 (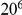), the cross-reactivity in our model is 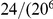), which is 1 in 2.7 million or 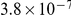.

### Immunogenic/non-immunogenic pMHCs

Four sets of pMHCs were obtained for which the immunogenicity had been determined previously. The first set of HIV-1 derived peptides presented on HLA-A02 was determined by Frankild et al. [9], who predicted which HIV-1 peptides were presented on HLA-A02 and then defined the ones as immunogenic if there was at least one report of a T-cell response in a patient in the Los Alamos Database. Because HIV-1 responses for the most frequent HLA-A*02 molecule are studied extensively, we defined all other peptides as non-immunogenic. Thus, 33 immunogenic and 54 non-immunogenic HIV-1 derived peptides were defined using this strategy. The second set is derived from Assarsson et al. [10], who tested the immunogenicity of vaccinia derived peptides in a humanized mouse-system expressing HLA-A*02. We classified the 9 mers shown to be naturally processed and immunogenic (termed “Dominant” and “Subdominant”) as immunogenic peptides, and non-immunogenic peptides (termed “Negative”) were classified as such. This resulted in the selection of 18 immunogenic and 26 non-immunogenic vaccinia derived peptides. The third data set is derived from Perez et al [37], who measured the T-cell response in HIV-1 patients to a set of HIV-1 peptides. The patients were HLA class I genotyped [37]. We only considered responses to 9mer peptides with a predicted binding affinity of less than 500 nM, to only one of the patients HLA-A and HLA-B molecules. Binding predictions were done with NetMHCpan-2 [46]. The virus in every patient was sequenced by Perez et al. [37], and we excluded all T-cell responses in which the peptide that was used for testing the T-cell response was not encoded by the viral genome. Only peptides presented on HLA molecules other than HLA-A*0201 were selected since HLA-A*0201 presented HIV-1 peptides were already compared in the data set derived from Frankild et al [9]. Peptide-HLA combinations with only negative T-cell responses measured by Perez et al. were classified as non-immunogenic (n = 13), all other peptide-HLA combinations were classified as immunogenic (n = 9). The fourth data set was derived from the IEDB [38], by downloading all entries that describe a T-cell response assay to a 9mer peptide presented on one of the HLA molecules in our test set, performed in a human subject upon infection. Only peptide-HLA combinations in which the predicted binding affinity was less than 500 nM were considered. Furthermore, we required that the assayed T-cells were not re-stimulated in vitro, and that the peptide was used in the T-cell response assay. Peptide-HLA combinations were classified as immunogenic if a “Positive(-High)” or “Positive-Low” T-cell response was measured, and classified as non-immunogenic if the T-cell response was always reported to be “negative”. We were able to classify more than 20 immunogenic and 20 non-immunogenic peptides only for HLA-A*0201 (i.e. 197 immunogenic and 592 non-immunogenic peptides).

### Additional anchor selectivity

For all HLA molecules, we predicted the binding of 1.000.000 random peptides with equal amino acid frequencies using NetMHC-3.2 and the thresholds described above. The Shannon entropy was determined per position on the predicted binders, per HLA molecule, and used as a measure of selectivity. Based on this selectivity, the six least specific positions were determined for each HLA molecule to use in the “allele specific” analysis of degenerate self/nonself overlaps (Table S2). Additional anchor selectivity was calculated as the sum of the entropy at the non-anchor positions (P1 and P3–8), per HLA molecule. An HLA molecule was defined to have additional anchors if the additional anchor selectivity was larger than 25% of the sum of entropy at all positions (P1–9) for an HLA molecule.

### Analyzing TCR-pMHC structures

Structures of HLA-I-9mer-TCR-complexes were downloaded in August 2011 from the PDB-database (www.pdb.org [30]). After redundancy reduction we selected nine structures for further analysis: 1AO7, 1BD2, 1LP9, 1MI5, 2ESV, 3GSN, 3KPR, 3O4L and 2F53 [49]–[57]. The selected structures consist of HLA-A*02 (n = 6), HLA-B*08, HLA-B*44 and HLA-E molecules. Per peptide position the number of TCR contacts was determined as the number of TCR amino acids within a 5.0 Å distance. For each structure, we determined per peptide position the fraction of TCR contacts relative to all peptide-TCR contacts in that structure. Boxplots of these fractions are shown in Figure 3.

### Statistics

Statistical tests were performed using the stats-package from the scipy-module in Python. A Permutation test was also done in Python, using the shuffle function in the random-package from the numpy-module, to identify human proteins that have more than expected peptides that overlap with viruses or bacteria. The permutation test was performed as follows: per human protein, we counted the number of viruses or bacteria that overlap with a 9mer peptide in this protein. These counts were normalized by the length of the protein, i.e. the number of overlapping viruses or bacteria was divided by the protein length. In 1000 permutations, per human protein a number of overlapping viruses or bacteria was drawn based on the expected fraction of overlaps and given the protein length. If the actual number of overlaps was higher than the number in all 1000 permutations, the human protein was selected as a protein with a significantly high number of viral or bacterial overlaps.

A similar analysis was performed to identify proteins with more than expected HLA-B*5401 ligands. First, per protein the number of HLA-B*5401 binding peptides was predicted as described above. Next, this prediction was compared in 1000 permutations where a number of binding peptides was drawn based on the specificity of HLA-B*5401 (i.e. 2.3% as described above). If the actual number of binding peptides was higher than the number in all 1000 permutations, the protein was selected as a protein with a significantly high number HLA-B*5401 ligands.

## Supporting Information

Figure S1 **Self/nonself overlaps based on non-anchor positions.** For different HLA molecules, the exact self/nonself overlap was determined based on non-anchor positions (P1 and P3–8). The average overlap was 0.4%.
(PDF)

Figure S2
**The self/nonself overlap of identical and non-identical overlaps versus the binding specificity.** The precise overlap of all peptide positions (P1–9, left figure, y-axis), and the degenerate overlap of the T-cell recognized middle positions (P3–8, right figure, y-axis), as well as the fraction of presented self peptides (both figures, x-axis) for each HLA molecule. The overlap and binding fraction were determined for every HLA molecule using scaled (in red) and fixed (in blue) binding thresholds. As discussed in the main text, a larger number of presented self peptides will lead to a larger chance of finding a self/nonself overlap. However, this does not hold if the self and nonself peptides are required to be identical to overlap (left figure), in which case the binding affinities of the self and nonself peptide are the same, and the chance of having an overlap with self depends solely on the presence of that peptide in the self proteome. Since the overlap is based on presented nonself peptides, if the self peptide is present it must be presented given the identical binding affinities. The correlation of overlap versus binding specificity illustrate this difference between identical and non-identical overlaps, data points obtained under the fixed threshold (in blue) were used in a Spearman Rank test (right figure: correlation = 0.89, p
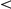
0.001; left figure: correlation = 0.25, p = 0.20).(PDF)

Table S1
**Human proteins that overlap with more than expected bacteria and viruses.** Human proteins that overlap at the 9mer level with a significantly large number of viruses or bacteria were analyzed using the on-line annotation analyzer DAVID [26], [27]. For the 10 most enriched non-redundant annotation clusters, the category encompassing most proteins is shown. All categories were significantly enriched (p
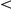
10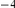).(PDF)

Table S2
**Degenerate T-cell recognition leads to high self/nonself overlaps under various conditions.** The self/nonself overlap was determined for the HLA molecules in our set (see Methods) and the average of the set is shown per cell. In the six columns on the right, the positions are shown on which the overlap is based, in the “allele specific” case the 6 least specific positions (see Methods) were selected for every HLA molecule, to allow for a-typical anchors in other positions. Overlaps were determined as “exact”, i.e. every position should be identical, or as degenerate (all other columns), i.e. with 1 or 2 substitutions being allowed to mimic the degeneracy of T-cell recognition (see Methods). The matrix that was used for determining amino acid similarity is shown in brackets. Overlaps with 100% or (a randomly chosen) 50% of the human proteome are shown in different rows. 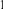
NetMHCpan-2 predictions (see Methods). 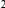
SMM binding predictions (see Methods). 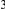
The analysis was done only for HLA-A*0101, HLA-A*0201, HLA-A*0301, HLA-B*0702, HLA-B*0801 and HLA-B*3501. 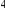
Using a fixed binding threshold of 500 nM instead of a scaled threshold. 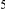
Amino acid substitutions were allowed next to each other.(PDF)
