# Supplementary material for: Degenerate T-cell Recognition of Peptides on MHC Molecules Creates Large Holes in the T-cell Repertoire
Source: PLoS Comput Biol. 2012 Mar 1;8(3):e1002412. doi: 10.1371/journal.pcbi.1002412 (PMC3291541; doi:10.1371/journal.pcbi.1002412)
Supplement: Figure S1 — Self/nonself overlaps based on non-anchor positions. For different HLA molecules, the exact self/nonself overlap was determined based on non-anchor positions (P1 and P3–8). The average overlap was 0.4%. (PDF) [file pcbi.1002412.s001.pdf]

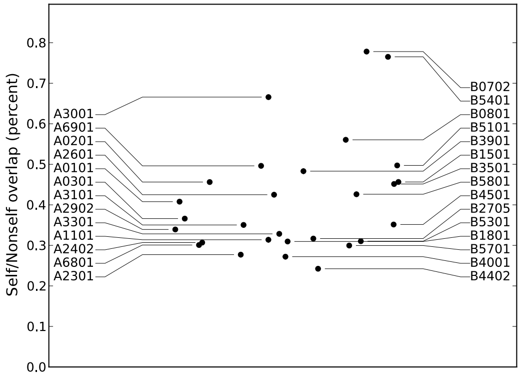

Figure S1: Self/nonself overlaps based on non-anchor positions. For different HLA molecules, the exact self/nonself overlap was determined based on non-anchor positions (P1 and P3-8). The average overlap was 0.4%.
