# Supplementary material for: Degenerate T-cell Recognition of Peptides on MHC Molecules Creates Large Holes in the T-cell Repertoire
Source: PLoS Comput Biol. 2012 Mar 1;8(3):e1002412. doi: 10.1371/journal.pcbi.1002412 (PMC3291541; doi:10.1371/journal.pcbi.1002412)
Supplement: Figure S2 — The self/nonself overlap of identical and non-identical overlaps versus the binding specificity. The precise overlap of all peptide positions (P1–9, left figure, y-axis), and the degenerate overlap of the T-cell recognized middle positions (P3–8, right figure, y-axis), as well as the fraction of presented self peptides (both figures, x-axis) for each HLA molecule. The overlap and binding fraction were determined for every HLA molecule using scaled (in red) and fixed (in blue) binding thresholds. As discussed in the main text, a larger number of presented self peptides will lead to a larger chance of finding a self/nonself overlap. However, this does not hold if the self and nonself peptides are required to be identical to overlap (left figure), in which case the binding affinities of the self and nonself peptide are the same, and the chance of having an overlap with self depends solely on the presence of that peptide in the self proteome. Since the overlap is based on presented nonself peptides, if the self peptide is present it must be presented given the identical binding affinities. The correlation of overlap versus binding specificity illustrate this difference between identical and non-identical overlaps, data points obtained under the fixed threshold (in blue) were used in a Spearman Rank test (right figure: correlation = 0.89, p0.001; left figure: correlation = 0.25, p = 0.20). (PDF) [file pcbi.1002412.s002.pdf]

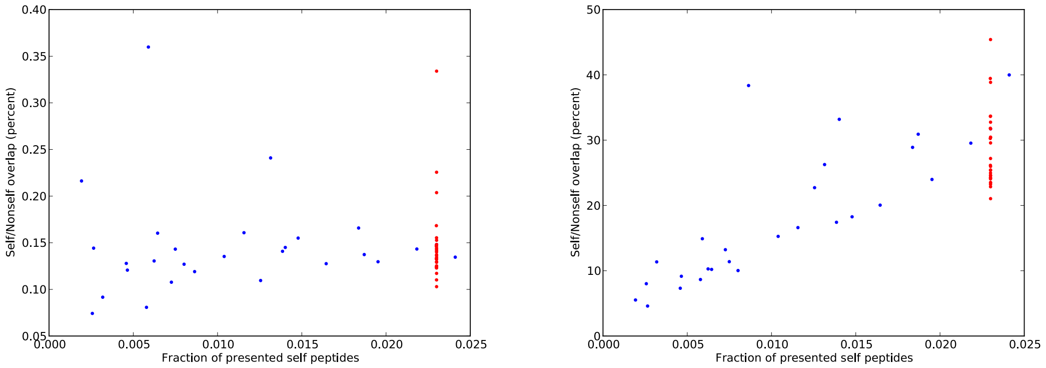

Figure S2: The self/nonself overlap of identical and non-identical overlaps versus the binding specificity. The precise overlap of all peptide positions (P1-9, left figure, y-axis), and the degenerate overlap of the T-cell recognized middle positions (P3-8, right figure, y-axis), as well as the the fraction of presented self peptides (both figures, x-axis) for each HLA molecule. The overlap and binding fraction were determined for every HLA molecule using scaled (in red) and fixed (in blue) binding thresholds. As discussed in the main text, a larger number of presented self peptides will lead to a larger chance of finding a self/nonself overlap. However, this does not hold if the self and nonself peptides are required to be identical to overlap (left figure), in which case the binding affinities of the self and nonself peptide are the same, and the chance of having an overlap with self depends solely on the presence of that peptide in the self proteome. Since the overlap is based on presented nonself peptides, if the self peptide is present it must be presented given the identical binding affinities. The correlation of overlap versus binding specificity illustrate this difference between identical and non-identical overlaps, data points obtained under the fixed threshold (in blue) were used in a Spearman Rank test (right figure: correlation=0.89, $p<0.001$; left figure: correlation=0.25, $p=0.20$).
