# Supplementary material for: Degenerate T-cell Recognition of Peptides on MHC Molecules Creates Large Holes in the T-cell Repertoire
Source: PLoS Comput Biol. 2012 Mar 1;8(3):e1002412. doi: 10.1371/journal.pcbi.1002412 (PMC3291541; doi:10.1371/journal.pcbi.1002412)
Supplement: Table S1 — Human proteins that overlap with more than expected bacteria and viruses. Human proteins that overlap at the 9mer level with a significantly large number of viruses or bacteria were analyzed using the on-line annotation analyzer DAVID [26], [27]. For the 10 most enriched non-redundant annotation clusters, the category encompassing most proteins is shown. All categories were significantly enriched (p10). (PDF) [file pcbi.1002412.s003.pdf]

Human proteins overlapping with bacteria (n=2055; 1726 found in DAVID)		
Cluster	Description	Number of proteins
1	nucleotide binding	525
2	mitochondrion	276
3	ATPase, AAA+ type, core	95
4	ATPase activity	137
5	membrane-enclosed lumen	281
6	NAD(P)-binding domain	72
7	monosaccharide metabolic process	62
8	magnesium ion binding	100
9	fatty acid metabolic process	54
10	mitochondrial part	160
Human proteins overlapping with viruses (n=722; 590 found in DAVID)		
Cluster	Description	Number of proteins
1	nucleus	299
2	transcription factor activity	116
3	membrane-enclosed lumen	112
4	regulation of transcription from RNA polymerase II promoter	85
5	RNA binding	58
6	pattern specification process	35
7	negative regulation of macromolecule metabolic process	51
8	non-membrane-bounded organelle	95
9	transcription factor binding	48
10	compositionally biased region: Arg/Ser-rich (RS domain)	10

Table S1. Human proteins that overlap with more than expected bacteria and viruses. Human proteins that overlap at the 9mer level with with a significantly large number of viruses or bacteria were analyzed using the on-line annotation analyzer DAVID [26,27]. For the 10 most enriched non-redundant annotation clusters, the category encompassing most proteins is shown. All categories were significantly enriched (p<0.0001).
