# Supplementary material for: Degenerate T-cell Recognition of Peptides on MHC Molecules Creates Large Holes in the T-cell Repertoire
Source: PLoS Comput Biol. 2012 Mar 1;8(3):e1002412. doi: 10.1371/journal.pcbi.1002412 (PMC3291541; doi:10.1371/journal.pcbi.1002412)
Supplement: Table S2 — Degenerate T-cell recognition leads to high self/nonself overlaps under various conditions. The self/nonself overlap was determined for the HLA molecules in our set (see Methods) and the average of the set is shown per cell. In the six columns on the right, the positions are shown on which the overlap is based, in the “allele specific” case the 6 least specific positions (see Methods) were selected for every HLA molecule, to allow for a-typical anchors in other positions. Overlaps were determined as “exact”, i.e. every position should be identical, or as degenerate (all other columns), i.e. with 1 or 2 substitutions being allowed to mimic the degeneracy of T-cell recognition (see Methods). The matrix that was used for determining amino acid similarity is shown in brackets. Overlaps with 100% or (a randomly chosen) 50% of the human proteome are shown in different rows. NetMHCpan-2 predictions (see Methods). SMM binding predictions (see Methods). The analysis was done only for HLA-A*0101, HLA-A*0201, HLA-A*0301, HLA-B*0702, HLA-B*0801 and HLA-B*3501. Using a fixed binding threshold of 500 nM instead of a scaled threshold. Amino acid substitutions were allowed next to each other. (PDF) [file pcbi.1002412.s004.pdf]

		Recognized peptide positions					
	Self percentage	P1-9 (complete)	P1 and P3-8 (non-anchor)	P3-8 (middle)	allele specific	P3-9	P2-8
Exact	100	0.15%	0.41%	2.7%	2.5%	0.92%	0.8%
	50	0.085%	0.24%	1.6%	1.5%	0.54%	0.48%
Degenerate (PMBEC)	100	0.71%	5.2%	29%	28%	12%	10%
	50	0.44%	3.2%	19%	19%	7.8%	6.6%
Degenerate (Blosum 62)	100	0.86%	6.8%	36%	35%	16%	13%
	50	0.52%	4.1%	25%	24%	10%	8.5%
Degenerate (Blosum 50)	100	0.98%	7.9%	40%	40%	18%	15%
	50	0.59%	4.9%	28%	28%	12%	9.8%
NetMHCpan[1,3] (PMBEC)	100	0.85%	7%	36%	35%	16%	13%
	50	0.52%	4.3%	26%	25%	10%	8.4%
SMM[2,3] (PMBEC)	100	0.89%	7.9%	38%	39%	16%	14%
	50	0.55%	4.9%	27%	28%	10%	8.9%
fixed[4] (PMBEC)	100	0.63%	3.5%	18%	18%	8.9%	7.4%
	50	0.4%	2.1%	12%	12%	5.6%	4.6%
Fully degenerate[5] (PMBEC)	100	0.94%	7.1%	35%	35%	17%	14%
	50	0.57%	4.3%	24%	24%	11%	8.7%

Table S2: Degenerate T-cell recognition leads to high self/nonself overlaps under various conditions. The self/nonself overlap was determined for the HLA molecules in our set (see Methods) and the average of the set is shown per cell. In the six columns on the right, the positions are shown on which the overlap is based, in the “allele specific“ case the 6 least specific positions (see Methods) were selected for every HLA molecule, to allow for a-typical anchors in other positions. Overlaps were determined as ”exact“, i.e. every position should be identical, or as degenerate (all other columns), i.e. with 1 or 2 substitutions being allowed to mimic the degeneracy of T-cell recognition (see Methods). The matrix that was used for determining amino acid similarity is shown in brackets. Overlaps with 100% or (a randomly chosen) 50% of the human proteome are shown in different rows. 1) NetMHCpan-2 predictions (see Methods). 2) SMM binding predictions (see Methods). 3) The analysis was done only for HLA-A*0101, HLA-A*0201, HLA-A*0301, HLA-B*0702, HLA-B*0801 and HLA-B*3501. 4) Using a fixed binding threshold of 500nM instead of a scaled threshold. 5) Amino acid substitutions were allowed next to each other.
